# PReS-FINAL-2148: Rheumates@work a cognitive behavioural internet based intervention promoting physical activity in children with juvenile idiopathic arthritis: preliminary results of a randomized clinical trail

**DOI:** 10.1186/1546-0096-11-S2-P160

**Published:** 2013-12-05

**Authors:** J Bos, W Armbrust, J Geertzen, P Sauer, P Dijkstra, M Van Brussel, J Cappon, O Lelieveld

**Affiliations:** 1 UMCG, Groningen, Netherlands; 2 WKZ UMCU, Utrecht, Netherlands; 3 Reade, Amsterdam, Netherlands

## Introduction

Juvenile Idiopathic Arthritis (JIA) is a chronic disease in which periods of active inflammation alternate with periods of inactive disease in an unpredictable way. Although impairments are most pronounced in children with disease activity, deficits like fatigue, decreased physical activity, low aerobic and anaerobic exercise remain impaired long after disease control is obtained. Exercise and physical activity (PA) can be seen as a type of behaviour. Therefore we expect that cognitive behavioural therapy (CBT) could be a successful approach to improve exercise capacity and PA levels in children with JIA. To increase PA levels in children with JIA an internet-based program has been developed. A pilot showed to be effective in improving PA and exercise capacity in children with JIA.

## Objectives

The aim of this multicenter study is to explore the efficacy of an internet based cognitive behavioural intervention Rheumates@work on PA and exercise capacity.

## Methods

We performed a randomized controlled trial. Patients with JIA aged 8-12 year, with access to internet were selected for this study.

PA was measured with a 7-day activity diary and an Actical accelerometer. PA level was categorized by time spend on moderate to vigorous PA and the number of days with 1 hour of moderate to vigorous PA. Aerobic exercise capacity was assessed by the Bruce treadmill test expressed by walking time. Disease activity was assessed by using the JIA core set. Adherence was electronically monitored. Patients with low physical activity defined as equal to or less than three days of one hour of moderate to vigorous PA or with a low exercise capacity defined as less than P5 on the Bruce treadmill test were included.

## Results

Out of 83 selected patients, 49 eligible patients were included and randomized in the intervention (n = 28) and control waiting list group (n = 21). Adherence was good 26 out of 28 patients (93%) completed the program. The intervention group improved significantly in exercise capacity (p.01), and in number of minutes spend on vigorous activity (p.00). The control group did not improve significant. Disease activity did not increase in both groups.

## Conclusion

Preliminary results show that the internet based cognitive behavioural program rheumates@work was effective in improving exercise capacity and stimulated the patients to be more vigorously active. Rheumates@work is safe to administer.

## Disclosure of interest

None declared.

